# 

**Published:** 1842-04-23

**Authors:** 


### ☞ TO SUBSCRIBERS.

In answer to inquiries, we beg leave to state, that we prefer, in all cases, that arrearages due us should be remitted *by mail* at our risk. Bank notes current in the States in which subscribers reside are received, when Philadelphia paper is not procurable, and postmasters are by law authorized to frank remittances.

Published every Saturday by J. C. TURNPENNY, N. E. corner of Spruce and Tenth streets.

*Price of Subscription*—Three Dollars a year, or two copies for Five Dollars to the same post office, always in advance. Subscriptions must commence with the beginning of the annual volume. Letters to be addressed, post free, to the Editors of the Medical Examiner.

*Rates of Advertising in the Medical Examiner*—For one insertion of a square of twelve lines, one dollar, and for each additional insertion, half a dollar.

